# Neuroendocrine Carcinoma of the Larynx and Pharynx: A Clinical and Histopathological Study

**DOI:** 10.3390/cancers13194813

**Published:** 2021-09-27

**Authors:** Primož Strojan, Robert Šifrer, Alfio Ferlito, Cvetka Grašič-Kuhar, Boštjan Lanišnik, Gaber Plavc, Nina Zidar

**Affiliations:** 1 Department of Radiation Oncology, Institute of Oncology Ljubljana and Faculty of Medicine, University of Ljubljana, 1000 Ljubljana, Slovenia; gplavc@onko-i.si; 2 Department of Otorhinolaryngology and Cervicofacial Surgery, University Medical Centre Ljubljana and Faculty of Medicine, University of Ljubljana, 1000 Ljubljana, Slovenia; robert_sifrer@hotmail.com; 3 Coordinator of the International Head and Neck Scientific Group, 35100 Padua, Italy; profalfioferlito@gmail.com; 4 Department of Medical Oncology, Institute of Oncology Ljubljana and Faculty of Medicine, University of Ljubljana, 1000 Ljubljana, Slovenia; cgrasic@onko-i.si; 5 Department of Otolaryngology, Head and Neck Surgery, University Medical Center Maribor, 2000 Maribor, Slovenia; bostjan.lanisnik@ukc-mb.si; 6 Institute of Pathology, Faculty of Medicine, University of Ljubljana, 1000 Ljubljana, Slovenia; nina.zidar@mf.uni-lj.si

**Keywords:** neuroendocrine carcinoma, head and neck cancer, diagnosis, therapy, prognosis

## Abstract

**Simple Summary:**

Neuroendocrine carcinomas (NECs) of the head and neck are rare. The presented series of 20 patients with laryngeal and pharyngeal NECs is population-based and one of the largest published to date. We analyzed the treatment results according to the type of therapy and the role of various standard (synaptophysin-chromogranin-CD56, Ki-67, p16, HPV, and EBV) and some novel (INSM1 and PD-L1) neuroendocrine markers or potential prognosticators. The results indicate the following: (1) laryngeal and pharyngeal NECs accounted for 0.43% and 0.17% of the cases in the corresponding tumor groups, respectively; (2) neuroendocrine differentiation can be reliably determined by INSM1 immunohistochemistry; (3) the prognosis was determined by the nodal stage and TNM stage but not by the histological grade (which refers to moderately and poorly differentiated NECs); (4) except in well-differentiated NECs and early-stage (T1-2N0-1) moderately/poorly differentiated NECs, aggressive multimodal therapy is needed; and (5) the p16, HPV, and EBV statuses failed to show any prognostic value.

**Abstract:**

Neuroendocrine carcinomas (NECs) of the head and neck are rare and the experience scanty. The Cancer Registry of Slovenia database was used to identify cases of laryngeal and pharyngeal NECs diagnosed between 1995–2020. Biopsies were analyzed for the expression of standard neuroendocrine markers (synaptophysin, chromogranin, CD56), INSM1, Ki-67, p16, and PD-L1 (using the combined positive score, CPS). In situ hybridization for human papillomavirus (HPV) and Epstein–Barr virus (EBV) was performed. Twenty patients (larynx, 12; pharynx, 8) were identified. One tumor was well differentiated (WD), five were moderately differentiated (MD), and 14 were poorly differentiated (PD). Disease control was achieved solely by surgery in 4/4 MD/PD T1-2N0-1 tumors. Eight patients died of the disease, seven of which were due to distant metastases. All three traditional markers were positive in 11/17 NECs and the INSM1 marker in all 20 tumors. Two of fourteen p16-positive tumors were HPV-positive, but all three nasopharyngeal NECs were EBV-negative. Three tumors had CPSs ≥ 1. In conclusion, INSM1 was confirmed to be a reliable marker of neuroendocrine differentiation. Except in WD and early-stage MD/PD tumors, aggressive multimodal therapy is needed; the optimal systemic therapy remains to be determined. p16, HPV, and EBV seem to bear no prognostic information.

## 1. Introduction

Primary neuroendocrine carcinomas (NECs) of the head and neck (HN-NEC) are rare. Their exact incidence is not known, and only estimates are available for the most common subgroups of these tumors, such as small-cell NECs (SCNECs) or those growing in the larynx, representing 0.27% and 0.38% of the head and neck tumor cases [1,2]. In addition, changes in the HN-NEC nomenclature over time have further contributed to the ambiguity regarding these tumors: they have mainly affected the group of moderately differentiated (MD) HN-NECs, putting into question the relevance of older literature data [3,4]. For example, a large-cell NEC (LCNEC) was defined as an independent histological entity in 2005 and was traditionally not separated from other MD HN-NECs [5,6,7,8]; only since 2017 has LCNEC been formally classified in the poorly differentiated (PD) category [9].

Due to their sporadic occurrence, only reports of individual cases or small patients’ series covering longer periods are available, which does not allow for a comprehensive analysis of the diagnostic and/or prognostic significance of individual histological markers or treatment approaches [10,11]. The diagnosis of HN-NEC is challenging and requires strict adherence to the 2017 WHO classification criteria, including the use of a panel of immunohistochemical markers to correctly classify these tumors [9]. However, the utility of traditional neuroendocrine markers (synaptophysin, chromogranin, and CD56) can be limited due to their wide range of sensitivities (individual or combined, 50–80%) and expression in tumor types other than neuroendocrine neoplasms, as well as the uncertain fidelity of cytoplasmic staining (i.e., weak and/or focal reactivity) [12].

In the HN-NEC patients, the treatment and survival results correlate with the disease stage, which is primarily determined by the degree of tumor differentiation and has not significantly changed with advances in diagnostics and treatment (e.g., the introduction of modern radiotherapy (RT) techniques and systemic treatments). While the disease-specific survival (DSS) for the notoriously rare well-differentiated (WD) HN-NECs is excellent, fewer than 20% of patients with PD tumors survive for five years after diagnosis [10,13]. As the curative potential of local therapies (surgery and radiotherapy) is eventually limited to the very early stage of the disease, in the majority of patients, the addition of systemic therapy is indicated to counteract microscopic or clinically overt metastases. While most tumors display an initial response to platinum-based chemotherapy (ChT) regimens, resistance develops at early timepoints during the course of disease, resulting in limited prognosis [14,15]. Thus, reliable prognostic markers and new therapeutic options are needed to improve the outcomes.

The present study aimed to review the experience with laryngeal and pharyngeal NECs that were diagnosed in Slovenia between 1995 and 2020. Special emphasis was placed on the pattern of treatment failure and survival depending on the treatment received and the role of standard and certain novel histological markers for the diagnosis and prognosis of the disease.

## 2. Materials and Methods

### 2.1. Patients

The study included patients with diagnosed NEC of the larynx or pharynx in Slovenia between 1995 and 2020. Cases of NEC were identified from a computerized search of the Cancer Registry of Slovenia database (https://www.onko-i.si/eng/sectors/epidemiology-and-cancer-registry, accessed on 9 August 2021), which is the state’s central service for collecting and managing data on new cancer patients. For each case, paraffin blocks were referred to the Institute of Pathology at the Faculty of Medicine Ljubljana for a central review to confirm the diagnosis of NEC and for grading according to the 2017 World Health Organization Classification of Head and Neck Tumors [9]. A representative block was chosen for immunohistochemical and in situ hybridization studies. The medical records of identified patients were reviewed to collect information on the characteristics of patients and tumors, therapy, and disease outcome. The tumors were staged using the criteria of the International Union against Cancer (UICC) TNM staging system, 7th edition [16].

### 2.2. Histological and Molecular Analyses

Biopsy tissue samples were fixed in 10% buffered formalin for 24 h, embedded in paraffin, cut at 4 μm, stained with hematoxylin and eosin, and analyzed according to standard criteria. Some immunohistochemical analyses and in situ hybridization was performed as part of the standard diagnostic procedures, and some were performed during this study.

#### 2.2.1. Immunohistochemistry

For immunohistochemistry, we used commercially available antibodies presented in Table 1. Staining was performed in the BenchMark XT immunostainer (Ventana Medical Systems, Tuscon, AZ, USA). After antigen retrieval with Cell Conditioning 1 buffer (Ventana Medical Systems) at 95 °C for 30 min, the slides were incubated with primary antibodies against the antigens. The immunoreactivity was visualized using the iVIEW DAB Detection Kit (Ventana Medical Systems), according to the manufacturer’s instructions. Sections were counterstained with hematoxylin. Positive controls and negative controls omitting the primary antibodies were also included.

p16 immunohistochemistry was considered positive if strong and diffuse nuclear and cytoplasmic expression was found in at least 75% of the tumor [17].

PD-L1 immunohistochemistry was determined by using the combined positive score (CPS), defined as the number of PD-L1-staining cells (tumor cells, lymphocytes, and macrophages) divided by the total number of viable tumor cells, multiplied by 100. The specimen was considered to have PD-L1 expression if CPS ≥ 1 [18].

#### 2.2.2. In Situ Hybridization

The HPV E6/E7 mRNA transcripts were analyzed using the commercially available in situ hybridization RNAscope 2.0, an HPV HR7 kit (Advanced Cell Diagnostics, Hayward, CA, USA), and targeting the E6/E7 mRNA of the HPV types 16, -18, -31, -33, -35, -52, and -58. The reactions were performed following the manufacturer’s instructions. Briefly, 4–5 µm tissue sections were first incubated at 60 °C for 1 h, followed by deparaffinization and incubation with pretreatment reagent 1 for 10 min at room temperature, pretreatment reagent 2 for 15 min at 100 °C, and pretreatment reagent 3 for 30 min at 40 °C. The tissue sections were then incubated with an HPV probe cocktail for 2 h at 40 °C. The hybridization complexes were visualized using the Detection Kit ACD (Advanced Cell Diagnostics, Hayward, CA, USA). The sections were counterstained with hematoxylin. A positive reaction was defined as punctate brown staining in the nuclei and/or cytoplasm [19].

In situ hybridization for the Epstein–Barr virus (EBV) was performed using the INFORM EBER Probe and the Ventana BenchMark XT Ultra automatic immunostainer following the manufacturer’s recommendations (Ventana Medical Systems, Tucson, AZ, USA). EBER-ISH was performed with an ISH iView Blue Detection Kit. Protein removal and nucleic acid exposures used ISH protease 3 (#780-4149). Counterstaining was performed with Red Counterstain II. For heat-induced epitope retrieval (HIER), the treatment time for cell conditioning (CC2) was varied. A positive reaction was defined as blue staining in the nuclei.

### 2.3. Statistics

The results were analyzed using the PC SPSS (Release 27, SPSS Inc., Chicago, IL, USA) statistical package. All of the tests were two-sided, and the results were considered significant at a probability level of ≤5%.

Basic descriptive statistics are reported as median (range) values for numerical variables and as percentages for categorical variables. An unpaired Student’s *t*-test was used for comparing age between groups, and the Pearson chi-square test was employed for statistical comparisons for categorical data. In the case of expected parameter values of <5 in >20% of cells, Fisher’s exact test was used, which facilitates the analysis of smaller population sizes. The follow-up period and survival were calculated from the date of diagnosis to 30 April 2021 (close-out date), or an event (relapse or death), whichever came first. Local tumor control was defined as a complete and permanent eradication of the tumor in the treatment area. The patients who died of progression of locally persistent disease after initial therapy were considered to have a local/regional event-free interval of zero. The Kaplan–Meier product limit method was used for a statistical assessment of local failure-free survival (LFFS), distant metastasis-free survival (DMFS), NEC-relapse-free survival (NEC-RFS, locoregional and distant failure considered as an event), disease-specific survival (DSS, deaths from disease unrelated causes censored), and overall survival (OS, all deaths considered as events). The differences between the groups were compared using the log-rank test.

## 3. Results

### 3.1. Patients

Over a period of 26 years, 20 patients with NECs of the pharynx or larynx were identified. The majority were men (15, 75%), former or active smokers (15, 75%), and aged 26–87 years (median: 64 years). The origin of 12 (60%) tumors was the larynx, three primary tumors each originated in the nasopharynx and hypopharynx, and two originated in the oropharynx, which represented 0.43% of all the laryngeal tumors (International Classification of Diseases, 10th revision (ICD-10) code C32) and 0.17% of all the pharyngeal tumors (ICD-10 codes C01 and C09–C14) diagnosed in Slovenia during this period [20]. Ten (50%) patients presented with a regional disease, and one (5%) patient presented with systemic metastases (lung).

**Figure 1:**
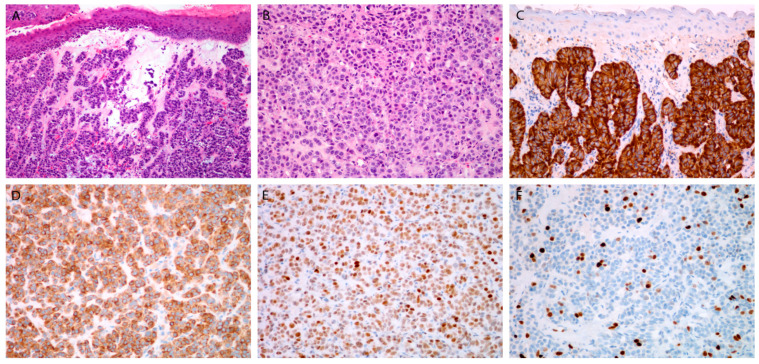
Moderately differentiated neuroendocrine carcinoma. Tumor beneath the surface epithelium, composed of uniform cell, with rare mitoses (**A**,**B**). Immunohistochemical staining for chromogranin (**C**), synaptophysin (**D**), INSM1 (**E**), and Ki-67 (**F**). Magnification 10 × 10 (**A**,**C**), 10 × 20 (**B**,**D**–**F**).

**Figure 2:**
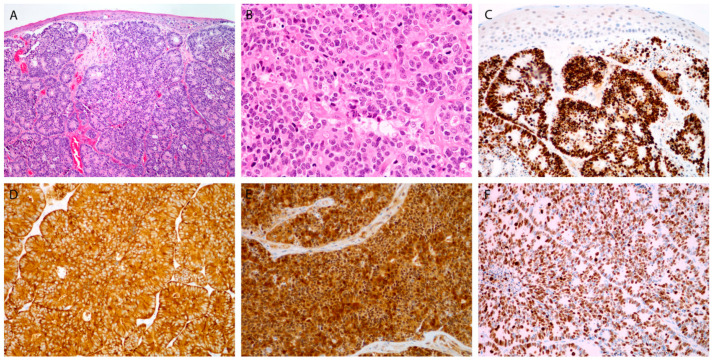
Large cell poorly differentiated neuroendocrine carcinoma. Tumor islands be-neath the surface epithelium, with rosette formation, composed of larger cells, with many mitoses (**A**,**B**). Immunohistochemical staining for INSM1 (**C**), synaptophysin (**D**), p16 (**E**), and Ki-67 (**F**). Magnification 10 × 10 (**A**,**C**), 10 × 20 (**B**,**D**–**F**).

**Figure 3:**
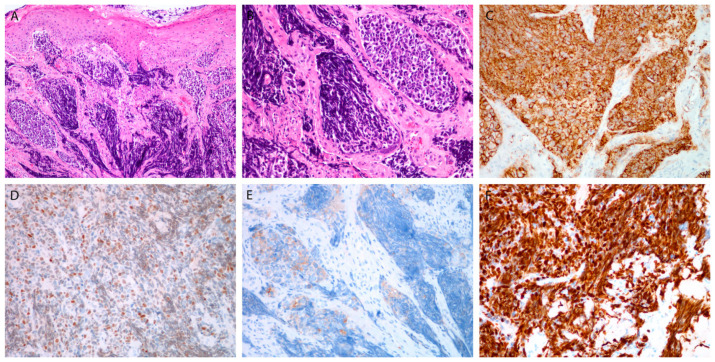
Small cell poorly differentiated neuroendocrine carcinoma. Tumor beneath the surface epithelium, composed of small hyperchromatic cells, with extensive crush artifacts (**A**,**B**). Immunohistochemical staining for CD56 (**C**), INSM1 (**D**), synaptophysin (**E**), and Ki-67 in almost all tumor cells (**F**). Magnification 10 × 10 (**A**,**C**), 10 × 20 (**B**,**D**–**F**).

Histologically, there were 1 WD NEC, 5 MD NECs (Figure 1), and 14 (70%) PD NECs (large cell, eight; small cell, six) (Figure 2 and Figure 3). One patient (Case 10) had a mixed laryngeal primary tumor consisting of LCNEC and squamous cell carcinoma. Patients with PD NECs more often had advanced disease at presentation (TNM Stage III-IV, WD + MD vs. PD: 2/6 vs. 12/14, *p* = 0.04) and were heavier smokers (defined as >15 pack-years (*N* = 17), WD + MD vs. PD: 0/6 vs. 8/11, *p* < 0.01). Early-stage disease (TNM Stage I–II) was more often diagnosed in females (females vs. males: 4/5 vs. 2/15, *p* = 0.01) and in patients with laryngeal primary tumors (laryngeal vs. other primaries: 6/12 vs. 0/8, *p* = 0.04). Patients with smaller primary tumors (Stage T1-2) were treated more frequently with upfront surgery (T1-2 vs. T3-4: 9/10 vs. 3/10, *p* = 0.02).

The detailed data on the characteristics of the patients and tumors, therapy, and survival are presented in Table 2.

### 3.2. Treatment

Ten patients had upfront surgery. Five also had postoperative (chemo)RT, and two of these (Cases 1 and 5) received no elective treatment to the neck. The other half received primary RT, either alone (*N* = 2) or in conjunction with ChT (*N* = 8).

In all cases, RT was conventionally fractionated (2 Gy/fraction, at five fractions/week), using a 6-MV linear accelerator photon beam and CT-based computer planning. Postoperatively, 60 Gy was delivered to the tumor bed and 64 Gy, to nodal metastases with confirmed extracapsular tumor spread, except in Patient 6, for whom the RT was prematurely terminated due to bacterial sepsis. In definitive settings, macroscopic disease was irradiated with 70 Gy and elective nodal regions were irradiated to an equivalent dose of 50 Gy. None of the patients received prophylactic cranial irradiation.

A concurrently administered ChT regimen consisted of 5–7 weekly applications (median: 6) of cisplatin at 30 mg/m^2^ (*N* = 2) or 40 mg/m^2^ (*N* = 4) or carboplatin at 1.5 AUC (*N* = 1); one patient received three cycles of cisplatin–etoposide combined with ChT (cisplatin at 75 mg/m^2^, Day 1; etoposide at 100 mg/m^2^, Days 1–3). In four patients, definitive (chemo)RT was preceded by three or four cycles of cisplatin–etoposide induction ChT (the same regimen as above).

### 3.3. Immunohistochemical and Molecular Analyses

Results of the immunohistochemical and molecular analyses are presented in Table 3.

#### 3.3.1. Markers for Diagnosis

Chromogranin and synaptophysin were positive in 14 (70%) and 17 (85%) tumors, respectively, whereas CD56 was positivity confirmed in all the analyzed tumors (100%) (Figure 1C,D, Figure 2C,D and Figure 3E). In 17 tumors, immunohistochemical staining for all three traditional markers was performed. Eleven of seventeen NECs (64.7%) were positive for all three markers; three tumors each stained positive for two or one of the markers (17.7%). All the studied NECs were positive for INSM1 (Figure 1E, Figure 2C and Figure 3D).

#### 3.3.2. Proliferative Markers

The Ki-67 tumor positivity ranged from <2% to 100% (median: 55%) (Figure 1F, Figure 2F and Figure 3F), and the mitosis number (per 10 high-power fields; not assessed in three PD tumors) ranged from 1 to 120 (median: 25). In WD and MD NECs, the percentage of Ki-67-positive cells was significantly lower than it was in PD tumors. A high degree of statistical correlation was found between the two proliferative markers (*p* < 0.01; RS = 0.75; 95% confidence interval (CI), 0.41–0.91). Ki-67 was particularly useful in small cell PD NECs in which mitotic figures were difficult to count due to crush artifacts (Figure 3F).

#### 3.3.3. Viral Markers

Fourteen tumors (70%) were p16-positive, but only two of them (12.5%) were found to be positive for high-risk HPV (Figure 2E). All of the p16-negative tumors were also HPV-negative. Of two oropharyngeal NECs, one was p16-positive, while both were negative for high-risk HPV. None of the three nasopharyngeal NECs exhibited a positive reaction for EBV.

#### 3.3.4. PD-L1

CPS was determined in 19 tumors, 3 of which had a CPS ≥ 1.

### 3.4. Treatment Outcome and Survival

Following the initial therapy, permanent local control was achieved in 17 (85%) treated tumors. One patient did not complete the primary treatment as planned (Case 6), and another patient (Case 20) with a PD NEC of the hypopharynx was left with a residual tumor upon the completion of concurrent chemoradiation (cCRT). In Patient 3, salvage neck dissection was successfully performed 79 months after surgery for an MD supraglottis NEC (without elective treatment to the neck) for an isolated regional recurrence, yielding an overall rate of ultimate local control of 90% (18/20).

Systemic metastases were diagnosed in seven patients (35%; MD NEC, 3; PD NEC, 4): in one patient at initial diagnosis and in six patients during the course of the disease (7 to 47 months after diagnosis; median: 9). All these patients had salvage treatment with different combinations of ChT, surgery, and/or RT and died of the disease within 0 to 31 months (median: 10) after metastatic spread was diagnosed.

Seven (35%) patients were alive and presented no evidence of disease on the close-out date, from 6 to 139 months (median: 60 months) after the diagnosis of the NEC: one patient with a WD tumor, two with MD tumors, and four with PD tumors. Eight (40%) patients died of the disease at 5–57 months (median: 12.5 months) post-diagnosis; systemic metastases were the cause of death in seven patients, and an uncontrolled primary tumor was the cause in one. Five (25%) deaths were due to intercurrent diseases that occurred within 3 to 114 months (median: 12 months) of NEC diagnosis. At the time of death, all these patients had no signs of active disease, including the one who died of sepsis during postoperative RT (Case 6).

The actuarial survival rates, with 95% CIs, at 2 and 5 years were, respectively, as follows: LFFS: 90% (77–100); DMFS: 63% (39–86) and 52% (25–79); NEC-RFS: 56% (34–79) and 47% (22–72); DSS: 77% (57–97) and 47% (20–74); OS: 64% (42–85) and 34% (12–57).

Upon univariate survival analysis, only the nodal stage and overall stage of the disease proved to be statistically important factors for predicting the DMFS (at 2/5 years, Stage N0 vs. N+: 89/71% vs. 29/29%, *p* = 0.04; Stage I–III vs. IV: 89/89% vs. 29/14%, *p* < 0.01), NEC-RFS (at 2/5 years, Stage N0 vs. N+: 89/71% vs. 23/23%, *p* = 0.02; Stage I–III vs. IV: 81/81% vs. 25/13%, *p* = 0.03), DSS (at 2/5 years, Stage N0 vs. N+: 100/69% vs. 51/25%, *p* < 0.01; Stage I–III vs. IV: 100/83% vs. 51/13%, *p* < 0.01), and OS (at 2/5 years, Stage N0 vs. N+: 89/61% vs. 40/10%, *p* < 0.01; Stage I–III vs. IV: 81/58% vs. 44/11%, *p* = 0.06). In addition, LC-NEC showed a tendency toward improved DSS compared to SC-NEC (at 2/5 years: 100/80% vs. 33/33%, *p* = 0.06). None of the patient-, disease-, or treatment-related factors tested affected locoregional control (Table S1).

In 11 patients with laryngeal MD or PD NEC, the corresponding rates at 2 and 5 years (and 95% CIs) were as follows: LFFS: 91% (74–100), DMFS: 62% (29–96), NEC-RFS: 57% (25–89), DSS: 87% (65–100) and 60% (24–96), and OS: 72% (44–99) and 49% (17–81). In this subgroup, the node negative neck status (Stage N0 vs. N+) also predicted improved 2/5 year DMFS (83/83% vs. 0/0%, *p* = 0.07), NEC-RFS (83/83% vs. 0/0%, *p* = 0.03), DSS (100/80% vs. 100/0%, *p* < 0.01) and OS (100/80% vs. 50/0%, *p* < 0.01), but not LFFS (Table S2). Neither grade (MD vs. PD) nor any of the other factors tested showed a statistically significant effect on the studied survival endpoints

## 4. Discussion

Due to its rarity, the available information on HN-NEC is limited to case reports or small series, which reflects the poor experience of clinicians with this disease. The present report on 20 patients with 12 laryngeal and 8 pharyngeal NECs is one of the largest single-institution series to date [21]. The central review of histological samples and extensive histopathological and molecular characterization of tumors minimized the risk of misdiagnosis and helped to clarify diagnostic and therapeutic dilemmas in this intriguing malignancy. In this context, despite its single-institutional and retrospective nature, the present study may represent solid ground on which relevant conclusions to be drawn.

The recognition of neuroendocrine differentiation is crucial for distinguishing NEC from squamous cell carcinoma of the head and neck. Due to inherent biological and clinical differences between the two groups of tumors, correct histological diagnosis has both therapeutic and prognostic implications [4]. The utility of traditional markers for the documentation of neuroendocrine differentiation, i.e., synaptophysin, chromogranin, and CD56, is limited due to several drawbacks: the imperfect sensitivity of only 50–80% (individual or combined) for high-grade NECs; cross-reactivity and, consequently, expression in other, non-neuroendocrine tumors of the head and neck; and the difficulty of interpreting the weak/focal reactivity of granular cytoplasmic staining. In addition, calcitonin is also common, though a non-specific marker of laryngeal NECs. Its secretion from laryngeal neuroendocrine cells is based on the embryological connection between a mesenchymal origin of the larynx and migration of the neural crest cells that later develop the characteristics of C cells. Furthermore, in rare cases, these patients even exhibit elevated serum levels of calcitonin, which presents a challenge in the differential diagnosis of medullary thyroid carcinoma [22]. On the other hand, INSM1 nuclear staining was found to be easier to interpret, with a superior 99% overall sensitivity across different neuroendocrine tumors of the head and neck and, specifically, with 95.8% sensitivity for PD NECs such as SC-NEC [12]. To the contrary, only 2.4% of non-neuroendocrine tumors stained positive for INSM1 in the study of Rooper et al., mainly histological types that showed reactivity with other neuroendocrine markers [12]. Our experience confirms a high prevalence of INSM1 positivity in laryngeal and pharyngeal NECs which corroborates previous findings that proposed its use as a first-line and stand-alone marker of neuroendocrine differentiation for head and neck tumors [12].

HN-NECs are rare malignancies, so the treatment recommendations are based on the results of small retrospective series or case reports published over several decades [10,11]. Due to the development of diagnostic and therapeutic methods and changes in the histological classification of these tumors over time, the survival results reported in pooled analyses should be interpreted with caution [3,4,5,6,7,8,9]. Our series is one of the largest published to date, although it includes only 20 patients treated over a 26-year period [21]. As proposed elsewhere, the incidence of NECs in Slovenia is negligible: these tumors represented only 0.43 and 0.17% of all the laryngeal and pharyngeal primary tumors diagnosed within the studied period [20]. Regardless of the treatment type and tumor grade, the locoregional control at 5 years post-therapy was excellent (90%), with only three failures: one was a non-responder to cCRT with an extensive cN3 neck mass (Case 20); the other was a late isolated regional recurrence successfully salvaged with surgery (Case 3); and the third was a case after R0 surgery who died of fatal sepsis during adjuvant radiotherapy (without any known disease, Case 6). As expected, the most common cause of death in our group was distant metastases. They occurred in six patients after the completion of primary treatment, in comparable proportions in the groups of MD (3/5) and PD (3/13) tumors (*p* = 0.27), and one patient had M1 disease at diagnosis (Case 19), resulting in no difference in outcomes between the two histologic groups. The 5-year DSS and OS in our series were 47 and 34% and did not differ with respect to the histopathological grade (WD + MD vs. PD), the primary tumor site (larynx vs. pharynx), or the treatment modality used (primary surgery vs. non-surgery) (Table S1). Given their wide 95% CIs, these figures are comparable to what has been reported in systematic reviews and cancer registry data analyses and pointed to a generally worse survival of HN-NECs compared with their squamous cell carcinoma counterparts [1,2,11,23,24].

In the present study, half of the patients were treated with upfront surgery, and the other half received definitive (chemo)RT. The surgical cases more often had smaller (T1-2) primary tumors (*p* = 0.02). Four of these patients with MD/PD NECs received no adjuvant (chemo)RT but remained free of disease at 6+ to 109+ months post-surgery. On the other hand, all but two elderly patients from the RT group had one form of ChT combined with irradiation, usually weekly platinum-based applications for the purpose of radiosensitization. High-dose induction or concurrent ChT (cisplatin/etoposide) directed to potential (micro)metastases was administered in five patients without obvious benefit, as systemic metastases developed/progressed during follow-up in three of them. Based on the above observations, we can only confirm that, besides in WD tumors, local treatment alone also has curative potential in early-stage HN-NECs of higher grades, assuring favorable disease control above clavicles and survival as already described by others [11,14,24]. However, an aggressive multimodal approach is needed for advanced disease, particularly in cases with extensive neck involvement, although no survival advantage of any of the treatment approaches (upfront surgery vs. non-surgery) was confirmed among our patients upon univariate analysis. An obvious diversity of the clinicopathologic features between the two therapeutic groups could play a role. Some large series also showed a lack of differences between various treatment scenarios in NEC [11,25].

The only prognosticators that were consistently of statistical significance for all the studied survival endpoints except the LFFS were the N-stage and the overall disease stage. The presence of neck metastases and Stage IV disease predicted worse survival outcomes: they affected not only the occurrence of distant metastases, which inevitably lead to patient death, but also predicted worse OS (which reflects both cancer- and non-cancer-related deaths). Comparable conclusions have also been reached in other, larger studies [11,24,25]. Currently, no effective systemic therapy is available: as mentioned above, three out of five of our patients who received cisplatin/etoposide ChT died of distant metastases. Though an encouraging initial effect of platinum-based ChT was found by Baker et al., the responses were typically not durable, and 80% of their patients ultimately succumbed to distant metastases [14]. According to the literature, a median progression-free survival of 4–9 months and an OS of 10–19 months can be expected in extensive/recurrent extrapulmonary PD NEC after cisplatin/etoposide-base ChT [15,26]. Unfortunately, other chemotherapeutic agents as well as specific schedules (e.g., metronomic ChT) employed in advanced/progressive NECs yield similarly unsatisfactory outcomes [27,28,29,30].

As an alternative, PD-1/PD-L1 checkpoint inhibitors, which are playing an increasingly important role in the treatment of metastatic squamous cell carcinoma of the head and neck, also appear to show some activity in a PD NEC, particularly when used in combination [18,31,32,33,34]. The PD-L1 expression in our patients and in the study by Bahr et al. was negligible [35]; however, as demonstrated by Ozdirk et al. in a small series of neuroendocrine neoplasms of different origin but also in other histological types, patients with negative or low PD-L1 expression on tumor cells may still derive some clinical benefit from anti-PD-L1 treatment [34,36]. Additional information is expected from studies exploring potential therapeutic strategies based on shared genomic alterations of NECs irrespective of their sites of origin [37].

The importance of p16/HPV status for the prediction of treatment response and prognosis in patients with PD NEC is uncertain. The results of p16 IHC and HPV status determination showed that, indeed, most (but not all) p16-positive and HPV-positive cases originated from the oropharynx; however, in contrast to the case for squamous cell carcinoma, the p16 status in PD NEC cannot be used as a surrogate marker of HPV status due to its low specificity [38,39,40]. Namely, the frequently seen overexpression of p16 in LC/SC-NEC of the head and neck may also be due to a virus-independent Rb pathway dysregulation [41]. Our results, with a p16 positivity of 70% and only two HPV-positive tumors (both were laryngeal primary tumors, one of them was MD NEC), corroborate these findings. Regarding the prognostic role of HPV, Benzerdjeb et al. recently reported an association between the involvement of HPV and improved OS in a pooled series of 78 cases of a PD HN-NEC [42]. Since the extent of the disease was not included in the multivariate model which, in several analyses (including ours), has proved to be an important prognostic factor, the results of this study should be interpreted with caution. Furthermore, upon reviewing the literature specifically focused on oropharyngeal PD NEC, Sinno et al. found no survival advantage of HPV positivity, although without regard to the stage of disease [40].

The prognostic importance of EBV in NECs is even less established than that of HPV. Cai et al. reported on 12 cases of nasopharyngeal NEC, 9 being PD tumors [43]. Among four tumors tested for EBV, one SC-NEC was negative, and three LC-NECs were positive, with favorable responses to chemoRT observed in two of them. An additional EBV-negative case of SC-NEC of the nasopharynx was reported by Mesonella et al. [44]. In the present series, the EBV testing was limited to three nasopharyngeal tumors, and all were negative: an MD NEC case, an SC-NEC case who developed systemic metastasis early, and an LC-NEC case with complete tumor clearance after RT and who died free of disease 12 months post-therapy. Despite favorable responses to chemoradiation in three of four reported EBV-positive LC-NECs (3 literature cases and Case 14), the small number of EBV-tested cases and limited/unknown follow-up information do not allow conclusions about the prognostic role of EBV in nasopharyngeal LC-NEC to be drawn [43].

An interesting observation from the present study is the high degree of correlation between immunohistochemical Ki-67 positivity and the number of mitoses in tumor cells. In doubtful cases or when, for technical reasons, the number of mitoses cannot be determined, a substitute marker of mitotic activity, which is one of the criteria for distinguishing between PD and other types of NEC, would be desirable [9]. In this context, further studies on the relationship between the Ki-67 labeling index and the mitosis number are warranted. In addition, a better response to the immune-checkpoint blockade was found to be associated with a higher proliferation rate [34].

Our study has weaknesses, which are mostly due to its retrospective nature and limited number of studied cases. Due to the retrospective design, it is possible that the incidence of NEC in the population is higher than that calculated; the initial histological classification of individual cases could be incorrect, and these cases were therefore not properly registered in the Cancer Registry database. During the 26-year period, tremendous progress has been made, especially in diagnostics and RT treatment, which is inevitably reflected in the differences in the quality of the management of patients diagnosed and treated at different times. However, the small number of patients did not allow the implementation of multivariate statistics that would take into account the differences in the distribution of prognosticators across groups being compared. Another drawback to consider is the limited amount of tissue for some patients, which meant that certain analyses could not be performed. Moreover, a few biopsies were over 20 years old, with a possible antigen degradation, which could have influenced our immunohistochemical analyses, particularly those with a nuclear reaction, e.g., Ki-67 [45]. Finally, we did not address certain relevant topics, such as the incidence and prognostic significance of paraneoplastic syndromes in NEC patients, the issue of mixed tumors, and prophylactic brain irradiation (PBI) [46,47,48,49,50]. Regarding the last, none of our patients had PBI: only in patients with extensive MD nasopharyngeal NEC did brain metastases occur 47 months after chemoradiation, supporting the view that PBI does not appear to play a role in this disease [50].

## 5. Conclusions

HN-NEC is a rare disease, representing a diagnostic and therapeutic challenge. Immunohistochemical testing to INSM1 was shown to be highly prevalent in this series of laryngeal and pharyngeal NECs. This is in line with previous findings, proposing that it can replace the traditional panel approach for the recognition of neuroendocrine differentiation. With the exception of WD tumors and early-stage MD/PD tumors, in which local treatment ensures a high degree of disease control and good long-term survival outcomes, others require aggressive multimodal treatment, especially in the presence of extensive neck disease. Due to the propensity to distant spread, early and effective systemic therapy is crucial. However, an optimal systemic agent as well as a therapeutic scenario remain to be determined. The statuses of p16, HPV, and EBV (in nasopharyngeal primary tumors) seem to bear no prognostic information. Pooled multi-institutional analyses, retrospective and prospective, are warranted to properly address the existing dilemmas.

## Figures and Tables

**Table 1:** Overview of source and clone, dilution of the primary antibodies, and antigen retrieval methods used for immunohistochemistry.

Antigen	Clone	Manufacturer	Pretreatment	Dilution
CD56	MRQ-42	Cell Marque, Rocklin, CA, USA	CC1 60 min	Ready to use
Chromogranin	polyclonal	Dako, Glostrup, Denmark	CC1 60 min	1:1000
Cytokeratin AE1/AE3	AE1AE3	Novocastra, Wetzlar, Germany	CC1 60 min	1:50
INSM1	A-8	Santa Cruz Biotechnology, Santa Cruz, CA, USA	CC1 56 min	1:50
Ki-67	MIB-1	Dako, Glostrup, Denmark	CC1 60 min	1:50
p16	E6H4	Ventana, Tuscon, AR, USA	CC1 32 min	Ready to use
PD-L1	SP263	Ventana, Tuscon, AR, USA	CC1 56 min	Ready to use
Synaptophysin	MRQ-40	Cell Marque, Rocklin, CA, USA	CC1 64 min	Ready to use

**Table 2:** Characteristics of patients, tumors and treatment.

Patients			Tumors			PET-CT	Therapy				Outcome			
No.	Sex/Age (Years)	Smoking Status	Site	TNM	Grade/ Type	(Yes/No)	Modality	Surgery	ChT (Drug/Cycles)	RT (Gy/fx)	DFI (mos)	Recurrence	Salvage Therapy	Status (Months)
Larynx														
1	M/64	Former/ 15 py	SG	pT1cN0, M0	WD	No	S	TLM			46+	No		NED
2	M/60	Former/ 15 py	SG	pT2pN2B, M0 ECE+	MD	No	S→RT	SGL ND(l)		L:60/30 R:64/32	12	Skin	S, ChT, RT	DOD (26)
3	F/59	No	SG	pT1pN0, M0	MD	No	S	SGL ND(b)			79	Neck node	S	NED (109)
4	M/60	No	SG	cT2cN0, M0	MD	No	cCRT		CP/5	L:70/35 R:56/35	7	Gallblader, skin	S ChT	DOD (38)
5	F/87	No	SG	pT2cN0, M0	MD	Yes	S	T			6+	No		NED
6	F/64	Active/ 26 py	SG	pT2pN1, M0	PD/LC	No	S→RT	SGL ND(b)		L:52/26 R:52/26	0	No		DOC (3)
7	M/72	Former/ 20 py	SG	cT3cN0, M0	PD/LC	No	RT			L:70/35 R:50/25	114	No		DOC
8	M/74	No	SG	pT1pN2B, M0 ECE+	PD/LC	No	S→RT	SGL ND(r)		L:60/30 R:64/32	8	Liver, lung	ChT	DOD (13)
9	F/26	Active/ 10 py	SG	cT2cN0, M0	PD/SC	Yes	cCRT		CP + E/3	L:70/35 R:56/35	80+	No		NED
10	F/62	Active/ 40 py	SG	pT1pN0, M0	PD/LC +SCC	Yes	S	SGL ND(b)			60+	No		NED
11	M/74	Former/ 10 py	SG	pT2pN2C, M0 ECE+	PD/LC	No	S→cCRT	TLM ND(b)	CaP/5	L:60/30 R:63/30	5,5	No		DOC
12	M/71	Former/ 60 py	SG	cT3cN0, M0	PD/SC	Yes	cCRT		CP/7	L:70/35 R:56/35	27+	No		NED
Nasopharynx														
13	M/67	No	NP	cT4cN0, M0	MD	No	iC→cCRT		CP + E/3 CP/5	L:70/35 R:50/25	47	Brain	RT	DOD (57)
14	M/80	Active/n.s.	NP	cT3cN0, M0	PD/LC	Yes	RT			L:70/35 R:56/35	12	No		DOC
15	M/54	Active/ 45 py	NP	cN2cN3, M0	PD/SC	Yes	iC→cCRT		CP + E/3 CP/6	L:70/35 R:70/35	7,5	Bone marrow	No	DOD (8,5)
Oropharynx														
16	M/67	Active/n.s.	OP	pT1pN1, M0	PD/LC	No	S	T(l) ND(l)			32	No		DOC (32)
17	M/64	Former/ 20 py	OP	cT4AcN2A M0	PD/LC	No	iC→cCRT		CP + E/4 CP/6	L:70/35 R:70/35	139+	N0		NED
Hypopharynx														
18	M/55	Active/ 35 py	HP	pT3pN2B, M0 ECE+	PD/SC	No	S→RT	pPH ND(l)		L:60/30 R:64/32	10	Liver, spleen, retroperitoneal	N0	DOD (10)
19	M/53	Active/ 40 py	HP	cT3cN3, M1/lung	PD/SC	No	iC→RT		CP + E/4	L:70/35 R:70/35	6	Lung	ChT	DOD (12)
20	M/59	Active/n.s.	HP	cT3cN3, M0	PD/SC	No	cCRT		CP/6	L:70/35 R:70/35	0	Residual tumor	No	DOD (5)

No.—number; M—male; F—female; py—pack-year; SG—supraglottis; NP—nasopharynx; OP—oropharynx; HP—hypopharynx; WD—well differentiated; MD—moderately differentiated; PD—poorly differentiated; LC—large cell; SC—small cell; S—surgery; RT—radiotherapy; cCRT—concurrent hemoradiotherapy; iC—induction chemotherapy; TLM—transoral laser microsurgery; SGL—supraglottic laryngectomy; ND—nodal dissection; T—tumorectiomy, pPH—partial; pharyngectomy; (l)—left; (b)—bilateral; (r)—right; ChT—chemotherapy; CP—cisplatin; E—etoposide; CaP—carboplatin; L—locally; R—regionally; DFI—disease-free interval; mos—months; NED—no evidence of disease; DOD—died of disease; DOC—died of other causes. One Patient received 26 of the planned 28 fractions of postoperative radiotherapy due to the development of bacterial sepsis, which was also the cause of death.

**Table 3:** Results of immunohistochemical and molecular analyses.

Patient’s No.	Sex/Age (Years)	Grade/Type	Chromogranin	Synaptophysin	CD56	INSM1	Ki-67	p16	HPV	PD-L1 (CPS)	Mitoses (per 10 hpf)
Larynx											
1	M/64	WD	+	+	+	+	<2%	-	-	Not done ^1^	1
2	M/60	MD	+	+	Not done ^1^	+	5%	+	-	0	3
3	F/59	MD	+	+	+	+	5%	+	-	0	9
4	M/60	MD	+	+	Not done ^1^	+	15%	+	-	0	6
5	F/87	MD	+	+	+	+	5%	-	-	3	3
6	F/64	PD/LC	+	+	Not done ^1^	+	20%	+	-	0	95
7	M/72	PD/LC	-	-	+	+	20%	-	-	0	Not possible ^2^
8	M/74	PD/LC	+	+	+	+	70%	+	+	0	33
9	F/26	SC/SC	-	+	+	+	100%	+	+	0	Not possible ^2^
10	F/62	PD/LC + SCC	+	+	+	+	80%	+	-	5	40
11	M/74	PD/LC	+	+	+	+	90%	+	-	0	30
12	M/71	PD/SC	-	+	+	+	90%	-	-	0	Not possible ^2^
Nasopharynx											
13	M/67	MD	+	+	+	+	>2%	-	-	0	1
14	M/80	PD/LC	+	+	+	+	30%	+	-	0	20
15	M/54	PD/SC	+	+	+	+	70%	+	-	0	28
Oropharynx											
16	M/67	PD/LC	+	+	+	+	70%	-	-	0	120
17	M/64	PD/LC	-	-	+	+	80%	+	-	1	25
Hypophyrynx											
18	M/55	PD/SC	+	+	+	+	80%	+	-	0	29
19	M/53	PD/SC	-	-	+	+	80%	+	-	0	30
20	M/59	PD/SC	-	+	+	+	40%	-	-	0	21

HPV was assessed by in situ hybridization for mRNA E6/E7 HPV. In situ hybridization for EBV was performed in 3 patients with tumors in the nasopharynx; it was negative in all. Cytokeratin was positive in all cases. In situ hybridization for HPV 6/11 was negative in all cases. No.—number, M—male; F—female; WD—well differentiated; MD—moderately differentiated; PD—poorly differentiated; LC—large cell; SC—small cell; SCC—squamous cell carcinoma; CPS—combined positive score; hpf—high power field. ^1^ Not done because no tissue was left. ^2^ Not possible to assess number of mitoses because of crush artifacts.

## Data Availability

The datasets analyzed during the current study are available from the corresponding author on reasonable request.

## Supplementary Materials

The following are available online at https://www.mdpi.com/article/10.3390/cancers13194813/s1, Table S1: Univariate analysis of survival, all patients (*N* = 20), Table S2: Univariate analysis of survival, moderately and poorly differentiated neuroendocrine carcinoma of the larynx (*N* = 11).
