# Supplementary material for: Neuroendocrine Carcinoma of the Larynx and Pharynx: A Clinical and Histopathological Study
Source: Cancers (Basel). 2021 Sep 27;13(19):4813. doi: 10.3390/cancers13194813 (PMC8507659; doi:10.3390/cancers13194813)
Supplement: Supplementary file 1 [file cancers-13-04813-s001.zip › cancers-1355418-supplementary.pdf]

Supplementary Material: Neuroendocrine Carcinoma of the Larynx and Pharynx: A Clinical and Histopathological Study

Primož Strojan, Robert Šifrer, Alfio Ferlito, Cvetka Grašič-Kuhar, Boštjan Lanišnik, Gaber Plavc and Nina Zidar

Table S1. Univariate analysis of survival, all patients ($N = 20$).

Parameter	*N*	Local Failure-Free Survival		Distant Metastasis-Free Survival		NEC Relapse-Free Survival		Disease Specific Survival		Overall Survival	
		At 2/5 Yrs, %	*P*-Value	At 2/5 Yrs, %	*P*-Value	At 2/5 Yrs, %	*P*-Value	At 2/5 Yrs, %	*P*-Value	At 2/5 Yrs, %	*P*-Value
Age											
<64 yrs	9	89/89	0.48	38/38	0.13	33/33	0.10	56/33	0.11	56/33	0.77
≥64 yrs	11	91/91		88/58		80/53		100/57		72/33	
Gender											
Female	5	80/80	0.20	100/100	0.10	80/80	0.52	100/100	0.07	80/80	0.14
Male	15	93/93		54/36		50/34		72/30		69/21	
Smoking status											
Nonsmokers[1]	11	100/100	0.29	67/50	0.84	67/50	0.47	100/49	0.33	91/44	0.09
Smokers	9	78/78		57/57		44/44		50/50		33/22	
Pack-years[2]											
≤15	9	100/100	0.97	57/38	0.80	57/38	0.84	100/38	0.99	89/34	0.96
>15	8	88/88		57/57		50/50		57/57		50/50	
Primary site											
Larynx	12	92/92	0.95	67/67	0.33	61/61	0.43	100/65	0.09	83/54	0.18
Pharynx	8	88/88		57/29		50/25		50/25		38/13	
NEC grade											
WD+MD	6	100/100	0.84	60/30	0.58	60/30	0.57	100/30	0.99	100/50	0.35
PD	14	86/86		64/64		55/55		67/58		53/31	
PD NEC[3]											
Small cell	6	83/83	0.83	40/40	0.14	33/33	0.18	33/33	0.06	33/33	0.63
Large cell	8	88/88		83/83		73/73		100/80		63/38	
Ki-67 status											
≤40	10	80/80	0.08	71/48	0.78	57/38	0.47	89/40	0.97	69/31	0.80
>70	10	100/100		56/56		56/56		67/56		60/38	
Stage T											
1+2	12	92/92	0.72	56/56	0.91	51/51	0.77	89/53	0.63	74/37	0.94
3+4	8	88/88		71/48		63/42		63/42		50/33	
Stage N											
N0	10	100/100	0.36	89/71	0.04	89/71	0.02	100/69	<0.01	89/61	<0.01
N+	10	80/80		29/29		23/23		51/25		40/10	
Overall stage											
I-III	11	91/91	0.85	89/89	<0.01	81/81	0.03	100/83	<0.01	81/58	0.06
IV	9	89/89		29/14		25/13		51/13		44/11	
First treatment											
Surgery	10	90/90	0.36	57/57	0.84	51/51	0.75	86/57	0.74	69/34	0.70
No surgery	10	90/90		67/50		60/45		70/42		60/36	

N–Number of patients; yrs–years; WD–Well differentiated; MD–Moderately differentiated; PD–Poorly differentiated; NEC–neuroendocrine carcinoma. 1, Former smokers included; 2, Data not available in 3 patients ($N = 17$); 3, Patients with poorly differentiated NEC only ($N = 14$).

Table S2. Univariate analysis of survival, moderately and poorly differentiated neuroendocrine carcinoma of the larynx (N = 11).

Parameter	N	Local failure-free survival		Distant metastasis-free survival		NEC relapse-free survival		Disease specific survival		Overall survival	
		At 2/5 yrs, %	*P*-value	At 2/5 yrs, %	*P*-value	At 2/5 yrs, %	*P*-value	At 2/5 yrs, %	*P*-value	At 2/5 yrs, %	*P*-value
Age											
≤64 yrs	6	83/83	0.25	60/60	0.87	50/50	0.39	100/60	0.86	83/50	0.86
>64 yrs	5	100/100		67/67		67/67		100/67		80/53	
Gender											
Female	6	100/100	0.20	40/40	0.13	40/40	0.70	100/30	0.10	83/25	0.25
Male	5	80/80		100/100		80/80		100/100		80/80	
Pack-years[3]											
≤15	7	100/100	0.65	40/40	0.13	40/40	0.36	100/40	0.16	86/34	0.42
>15	4	75/75		100/100		75/75		100/100		75/75	
NEC grade											
MD	4	100/100	0.65	33/33	0.22	33/33	0.22	100/33	0.35	100/33	0.98
PD	7	86/86		80/80		68/68		100/80		57/57	
Ki-67 status											
≤40%	6	83/83	0.25	50/50	0.51	42/42	0.22	100/50	0.70	83/42	0.80
>70%	5	100/100		75/75		75/75		100/75		60/60	
Stage N											
N0	7	100/100	0.19	83/83	0.07	83/83	0.03	100/80	<0.01	100/80	<0.01
N+	4	75/75		0/0		0/0		100/0		50/0	
Overall stage											
I+II	5	100/100	0.86	75/75	0.62	75/75	0.62	100/75	0.28	100/75	0.09
III+IV	6	83/83		50/50		42/42		100/50		67/33	
First treatment											
Surgery	7	86/86	0.13	50/50	0.62	43/43	0.18	100/50	0.43	71/36	0.19
No surgery	4	100/100		75/75		75/75		100/67		100/67	

N–Number of patients; yrs–years; NEC–neuroendocrine carcinoma; MD–Moderately differentiated; PD–Poorly differentiated.

Publisher's Note: MDPI stays neutral with regard to jurisdictional claims in published maps and institutional affiliations.

© 2021 by the authors. Licensee MDPI, Basel, Switzerland. This article is an open access article distributed under the terms and conditions of the Creative Commons Attribution (CC BY) license (http://creativecommons.org/licenses/by/4.0/).
